# Exploring the knowledge of community‐based nurses in supporting parents of preterm babies at home: A survey‐based study

**DOI:** 10.1002/nop2.937

**Published:** 2021-05-19

**Authors:** Julia Petty, Lisa Whiting, Cathrine Fowler, Janet Green, Alison Mosenthal

**Affiliations:** ^1^ Children’s Nursing University of Hertfordshire Hatfield Hertfordshire UK; ^2^ University of Technology Sydney Australia; ^3^ School of Nursing College of Health and Medicine University of Tasmania Hobart Australia

**Keywords:** community‐based nursing, knowledge gap, preterm babies, supporting parents, tailored resources

## Abstract

**Aim:**

This study aimed to investigate the confidence levels, knowledge base and learning needs of community‐based nurses relating to the care of preterm babies and parents, to explore what education is required and in what format.

**Design:**

An online survey methodology was used.

**Methods:**

A 32‐item questionnaire was distributed via social media platforms to community‐based nurses in Australia.

**Results:**

Descriptive analysis was undertaken relating to knowledge base, confidence levels, previous training, learning and resource needs and barriers to education. It was deemed vital to expand confidence and knowledge in this area. Gaps in learning resources were identified and a need for more training in topics such as developmental outcomes, feeding, expected milestones, weight gain, growth trajectories and supporting parents. Online resources were the preferred format to teach key knowledge to community‐based health professionals, tailored to the specific features of preterm babies and support needs of parents.

## 1 INTRODUCTION

As survival rates of preterm babies have increased, provision of care and support in the home environment after discharge from hospital‐based neonatal care is becoming increasingly crucial (Fowler et al., 2019; Petty et al., 2018). The provision of ongoing support at home becomes the role of the primary care team which includes family doctors, allied health professionals and, crucially, nurses as a central member of the team. Globally, various terms are given to community nursing roles so, in the context of this study, the term “community‐based nurse” will be used throughout for consistency. Green et al. (2020) have outlined the difficulties associated with the transition home of the preterm baby and their family, and Sharma et al. (2018) have acknowledged how community visits are a vital consideration in supporting families once they are at home. In addition, the educational needs of community‐based nurses must be addressed to equip them with appropriate knowledge and skills to support these families. This is particularly essential given the high value parents place on the relationship with professionals for ongoing advice following discharge from the neonatal unit (Garcia & Gephart, 2013). These teams should therefore be knowledgeable about the ongoing needs of preterm babies. However, recent research has identified a perceived gap in healthcare knowledge and confidence relating to supporting the needs of parents with preterm babies (Petty et al., 2018; Petty, Whiting, et al., 2019). This paper reports on a study that followed on from said research, which aimed to explore confidence levels, knowledge base and learning needs of community‐based nurses relating to the care of preterm babies and parents, to explore what education is required and in what format.

## 2 BACKGROUND

Limited previous research exists on the perceived gap in health professionals’ knowledge and learning needs relating to supporting parents of preterm babies in community settings. Available literature appears to focus more on medical knowledge, for example with paediatricians where a lack of information relating to the challenges of managing small, preterm babies was highlighted (Hussey‐Gardner & Donohue, 2015). Einaudi et al. (2013) examined physicians’ knowledge in the management of children born extremely preterm, finding a need for information on methods of assessment to inform care. Powell et al. (2012) reported that 50% of obstetric professionals found answering parental questions regarding their preterm infants challenging. Parker et al. (2012) identified that doctors were unaware of the stress levels felt by parents of preterm babies after discharge and were subsequently unprepared to support them through discharge. Furthermore, Kuppala et al. (2012) and Boss and Hobbs (2013) highlighted a perceived lack of training for doctors in this speciality leaving them feeling unable to care for medically complex babies and their developmental issues.

Recent literature, however, on nursing knowledge relating to community‐based care for preterm babies and families is sparse. Some exist in other nursing fields such as adult care (Dahlke et al.,^2019^) and critical care (Williams & Parry, 2018). It has been found, for example that healthcare providers frequently do not have the knowledge and confidence to engage in meaningful conversations about palliative care (Harden et al., 2017) leading to significant knowledge gaps (Carvalho et al., 2020).

In child and neonatal healthcare specifically, the importance of nursing knowledge and recognition of gaps features in some literature from low‐resource countries such as Nepal, Kenya and India, respectively (Adhikari et al., 2016; Murphy et al., 2019; Sharma et al., 2018). Other literature on community health professional education exists but without any inclusion of the need to learn about preterm babies and their families (Appleton et al., 2014; Condon et al., 2015; Malone et al., 2016). Cordewener and Lubbe (2017) described nurses’ perceptions of skills required to perform effective preterm baby assessments. Thirteen semi‐structured interviews were conducted, and the themes identified included lack of skills and knowledge to conduct quality assessments. Formal and continuous development training needs in this area were also highlighted.

Moreover, the limited commentary on the importance of neonatal specific education (Petty et al., 2019) tends to focus more on clinical care rather than the emotional, support needs of parents within the community setting. Clearly however, knowledge acquisition and training in caring for and supporting parents of preterm babies are important, given what has been reported in previous research regarding the lack of specific knowledge on prematurity (Fowler et al., 2019; Green et al., 2020; Petty et al., 2018).

This current study, therefore, set out to address the above‐mentioned gap in research relating to knowledge needs of community‐based nurses in neonatal care. Furthermore, the research question for this current study has emerged from the findings of previous enquiry by the authors, a collaborative research team that explored the parental needs of preterm babies at home from both the UK and Australian perspective (Petty et al., 2018; Petty, Whiting, et al., 2019). Joint funding in the form of a small, pump‐priming research grant between two Higher Educations Institutions (HEIs) in both countries allowed this collaboration to happen. The UK arm of data has been reported and published (Petty, Whiting, et al., 2019): Based on interview and questionnaire data of a sample of UK‐based community health professionals (Health Visitors (HVs), community children's nurses and educators), three main themes emerged from the data analysis: development of prior knowledge; the importance of practice‐based learning; learning and training needs. Knowledge, skills and confidence levels in relation to caring for parents with preterm babies varied between individuals depending on their training and subsequent experiences. While transferable skills in supporting parents in the community were present, more education and training in the specific needs of preterm babies and parents were recommended. The study also concluded that tailored resources for community‐based nurses on the specific needs of the preterm baby would enhance provision of optimal support for parents. This current study focuses on the Australian component of the research, as a follow‐on study from the UK arm.

The aim of this current study was to explore perceived confidence and knowledge needs necessary to support parents of preterm babies at home, both key areas of interest that emerged from the above research. In addition, the study aimed to provide insight into other vital elements that could be used to plan future education for community‐based nurses; namely, specific resource requirements, desired topics, format for delivery and perceived barriers to education. The research question posed was: What are the confidence levels, knowledge and resource needs relating to how to support families of preterm babies at home in Australian community‐based nurses?

## 3 DESIGN

The study employed an online survey design. A survey is a series of pre‐defined questions intended to collect information from people, whether in person, online or by other media. Surveys are especially important when addressing topics that are difficult to assess using other approaches and usually rely on self‐reporting, for example behaviours, such as satisfaction, beliefs, knowledge, attitudes and opinions. Online surveys are increasingly used in health education research because of their low cost and relative speed (Parahoo, 2014; Phillip, 2017). In this case, such a design offered opportunities to access participants across a large geographical area (Harrison et al., 2020), highlighting the benefits of this methodology to provide access to groups and individuals who would be difficult, if not impossible, to reach through other channels.

## 4 METHODS

Surveys are instruments used to quantitatively evaluate subjective data. Through the addition of open‐ended questions, qualitative data can be obtained. Survey is a general term used to describe the collection of information and is often used interchangeably with questionnaire—a list of focused questions (Hammer, 2017). In addition to the advantages acknowledged above, the findings can also, importantly, be swiftly and readily quantified. The online survey format in this study used questions that were primarily quantitative in nature, but also included free‐text questions. Regmi et al. (2016) comment that there are three key advantages to the online questionnaire: respondents can take as long as they like to finish it; they do not need to complete it in one sitting, and they can undertake it at a time to suit them. Regmi et al. (2016) offer six components that need considering if an online questionnaire is to be successful (Table 1); each of these were duly addressed in our research. This was deemed important as, according to Dillman et al. (2014), response rates to internet‐based surveys tend to be low, compared with paper‐based ones; therefore, it was necessary to provide scrutiny and enhance validity of the tool used. The survey was piloted within the research team; however, it was not tested externally for validity, an identified limitation of the study.

**TABLE 1:** Components and application of a successful online questionnaire (adapted from Regmi et al., 2016)

Components of a successful online questionnaire	Application to this study
User‐friendly design	The questionnaire was carefully planned so that the layout was clear and uncluttered with concise instructions being included.
Selecting survey participants	An online questionnaire was selected as the potential participants were health professionals who had strong internet skills and a familiarity with this type of data collection.
Avoiding multiple responses	Care was taken to ensure that each participant did not complete the questionnaire more than once
Data Management	The questionnaire software offered a robust and reliable data management system
Ethical issues	Appropriate ethical approval was sought and granted. In addition, details about implied consent and confidentiality were included on the questionnaire. The participants were able to leave sections of the tool blank if they did not wish to answer a question.
Piloting	The questionnaire was piloted with the research team itself, and minor amendments were made.

The questionnaire (Table 2) designed for this survey comprised two main sections: Part 1 collected general demographic information and nature of the participant role (questions 1–11). Part 2 contained questions regarding confidence, knowledge, education, experiences caring for premature babies and learning needs.

**TABLE 2:** Survey—Providing care and support for parents of premature babies at home


Items 1–11, 13, 15, 17, 19–20, 24–26, 28–29 and 31–32 provided a drop‐down list to select an option (either single or multi‐choice). Items 12 and 18 were a Likert scale asking respondents to choose one answer from a scale of 1–5. Items 14, 16, 21, 22, 23, 27 and 30 provided a box to add open‐ended responses.
**PART 1** Confirmation of consent Confirmation of being a child / family nurse or not Confirmation of English‐speaking What is your present age? Please select your gender. What registered healthcare professional are you? Please select the number of years of experience that you have caring for babies of or less than 27 weeks gestation who are in infancy or older What basis are you employed on? How often do you come into contact with growing premature babies in your role? Please select the age groups which you most commonly provide care to. Do you provide care to any of the following groups?
**PART 2** 12. Please rate the following statements about your current knowledge & confidence, previous training and learning needs relating to the specific support needs of parents of growing premature babies. (1=completely disagree / 2=agree / 3=neutral / 4=agree / 5‐completely agree) My knowledge of the needs of the growing premature baby is adequate My knowledge of how to support parents is adequate I have sufficient confidence to effectively support the needs of the parents of growing premature babies 13. How much training have you previously had in the specific needs of growing premature babies and their parents? 14. Please state the nature of this training 15. Do you think that you need more training in the specific needs of growing premature babies? 16. Please indicate which training you require 17. What is the best way to deliver this training? 18. Please rate the following questions regarding your learning needs. (1=completely disagree / 2=agree / 3=neutral / 4=agree / 5‐completely agree) I need more knowledge to equip me to support parents of growing premature babies I need greater confidence to equip me to support parents of growing premature babies I have received adequate training in the specific support needs of parents of growing premature babies 19. What areas of knowledge specific to the growing premature baby do you require to better equip you to support parents? 20. What resources are required to support you to understand the specific needs of parents of growing premature babies 21. Please use the space below to add any further comments about what training and education you require in relation to supporting parents of premature babies. 22. Could you give an example of issues that ex premature babies face that you have experienced? 23. What do you think are the specific care needs of the parents of the growing premature baby? 24. Where do you get your information/educational needs about growing premature babies that require ongoing technical support (including oxygen, nasogastric/peg feeds, monitoring, tracheostomy, mechanical ventilation) at home? 25. As a part of your care, do you carry out routine psychosocial assessments of the parents of the growing premature baby? 26. Do you have any special educational resources for the parents of growing premature babies? 27. What do you know about both the short‐ and long‐term outcomes for babies 27 weeks’ gestation and less, regarding growth, development, expected and future health issues? 28. Do you feel that you are acting as part of a multidisciplinary team caring for the growing premature baby and their mother? 29. Overall, do you feel confident in your ability to care for the growing premature baby and their family? 30. Where do you get support from when you feel that you do not have the skills to provide care to a growing premature baby, or when it is a critical situation? 31. What do you think the barriers that yourself and other nurses face which may stop them from undertaking further education and professional development, relevant to the growing premature baby? 32. What communication have you received regarding caring for the extremely premature baby and their family?


### 4.1 Sample and setting

The goal of sampling in survey research was to obtain a sufficient sample that is representative of the population of interest (Ponto, 2015), (i.e. community‐based nurses). The inclusion criteria were as follows: Registered or Enrolled nurses currently practising in community nursing roles, visiting families and babies in Australia, who were English speaking. The nurses may or may not have had exposure to preterm babies and families, and this was an area of interest within the survey; see question 9, Table 2. Recruitment to complete this online survey was through professional Australian nursing associations social media (Facebook) groups (namely, Child and Family Nursing Association, Council of Children's Nurses New‐South‐Wales and Nurse Path). Permission was sought to distribute the survey from the group administrators who acted at gatekeepers, all approved in the ethics application. The survey was circulated for three months with repeated postings.

### 4.2 Participants

Seventy‐two participants took part in the survey and completed the demographic questions in part 1. The analytics report of survey data highlighted that only 42 (58%) participants completed *all* questions comprising both part 1 (demographics) and part 2 (confidence, knowledge and learning needs). Thirty (42%) respondents only completed part 1. The reason for this was unclear and may have been related to the survey design, acknowledged as another possible limitation. There was no mitigation built into the survey against respondents completing it more than once; however, there was no evidence in the responses of any repetition. The demographics from part 1 of the survey (*n* = 72) are summarized in Table 3. Question responses relating to the participants’ community‐based role from part 2 (*n* = 42) will be outlined in the Findings section.

**TABLE 3:** Participant demographics

**Current role** All community‐based nurses, 36 (84%) working currently as nurses visiting homes, 6 (16%) working in community medical centres as practice nurses
**Age** An even proportion across all age bands (20–29 / 30–39 / 40–49 / 50–59 / +60 years) ‐ 6–9 in each band
**Gender** All were female (42 / 100%)
**Profession** All trained nurses (42 / 100%).
**Work hours** 18 (43%) fulltime, 21 (50%) part‐time and 3 (7%) casual.
**Frequency of contact with preterm babies in the community** Daily 5 (11.6%) Few times a week 8 (18.6%) Weekly 9 (20 0.9%) Monthly 10 (25.6%) Every 3 months 2 (4.7%) Every 6 months 3 (7.0%) Yearly or less often 4 (9.3%) Never 1 (2.3%)
**The number of years of experience caring for babies born less than 27 weeks gestation who are in infancy or older** Highest proportion had less than a year (6 / 14%). An even proportion of experience across the remaining number of years from 2–35 years.
**The age of the babies and children seen in practice** Majority up to 6 months of age, then up to 1–2 years old, and less contact with children over 5 years.

### 4.3 Analysis

Descriptive statistics were used to present quantitative data in a manageable form. In a survey‐based research study, there are numerous items to report. Descriptive statistics help to group or aggregate and simplify large amounts of data in an organized way, ensuring that data with many responses can be represented as a simpler summary (Guetterman, 2019). This form of analysis was employed for each of the survey items (questions). They were reported within question groupings that were regarded as themes: nature of the role, confidence levels, existing knowledge, previous training, learning and resource needs and barriers to education. The text‐based responses that participants offered to the open‐ended questions were also grouped according to these same themes. This method of analysis uses a deductive approach; in this study, two of the researchers independently read through and coded the open‐text data according to the above pre‐determined themes (Young et al., 2020). Verification and agreement were undertaken within the research team. These open responses added further qualitative data to support the quantitative, descriptive analysis and enrich the analysis.

### 4.4 Ethics

The study received ethical approval through the authors’ University governance processes (protocol number ETH 18–2534). Potential ethical issues associated with the use of online surveys were all appropriately addressed; namely, those relating to Anonymity and data security (for example, secure transmission, contact details were not requested and secure storage of participant data online). Participants were only able to access the survey when they had ticked the box at the bottom of the study information section indicating they had read it and given their permission to be involved in the research. It was important to use approved reporting guidelines; in this study, Kelley et al.’s (2003) checklist for good practice in the conduct and reporting of survey research was used.

## 5 RESULTS

The findings from this point relate to the 42 respondents who completed the whole survey, both parts 1 and 2 (the remaining 30 did not complete both parts so these were disregarded). A summary of the data for each item (from Table 2) is provided with some extracts of any relevant supporting open responses from selected participants (P), as appropriate.

### 5.1 Nature of the role

**FIGURE 1:**
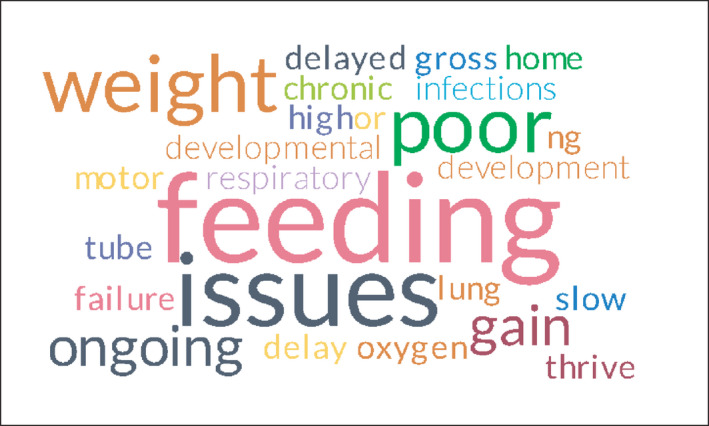
Most common clinical issues seen in the community setting with preterm babies (survey question 22)

**FIGURE 2:**
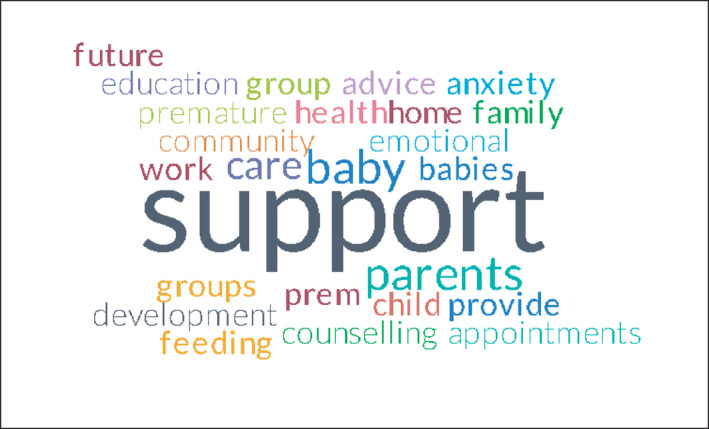
Most common care needs seen in the community setting with preterm babies (survey question 23)

In relation to the nature of the participants’ role, 29 (70%) felt they worked as part of a multi‐disciplinary team (MDT) with the remaining 13 (30%) responding that they did not work as part of the MDT or were “Unsure.” The issues facing them included babies with developmental problems, long‐term lung disease requiring oxygen, growth delay, poor weight gain and most frequently, ongoing feeding problems. The prevalence of these physical issues is depicted visually by a Word Cloud in Figure 1 that highlights the more common responses in larger text. In addition, the most common care needs are highlighted in Figure 2. Frequently identified was the need to support parents emotionally as a central part of the role with 21 respondents (50%) being involved in psychosocial assessments of parents. These assessments were extended to the need for improvements in the psychosocial care provided—“*the parents need to have better psychosocial care when they go home with their baby*” (P11). They received support from various colleagues, most commonly educators who play an essential role in the ongoing education and practice‐based training of community‐based nurses, outside the formal education received from HEIs. Self‐directed inquiry using web‐based sources of information was also an information source. Communication about preterm babies was generally done by discharge summaries for 25 (60%) with 5(12%) having no communication. For some participants, this communication was not identified as adequate requiring “… *more communication between the hospital and community‐based care*.” (P2).

### 5.2 Confidence

**FIGURE 3:**
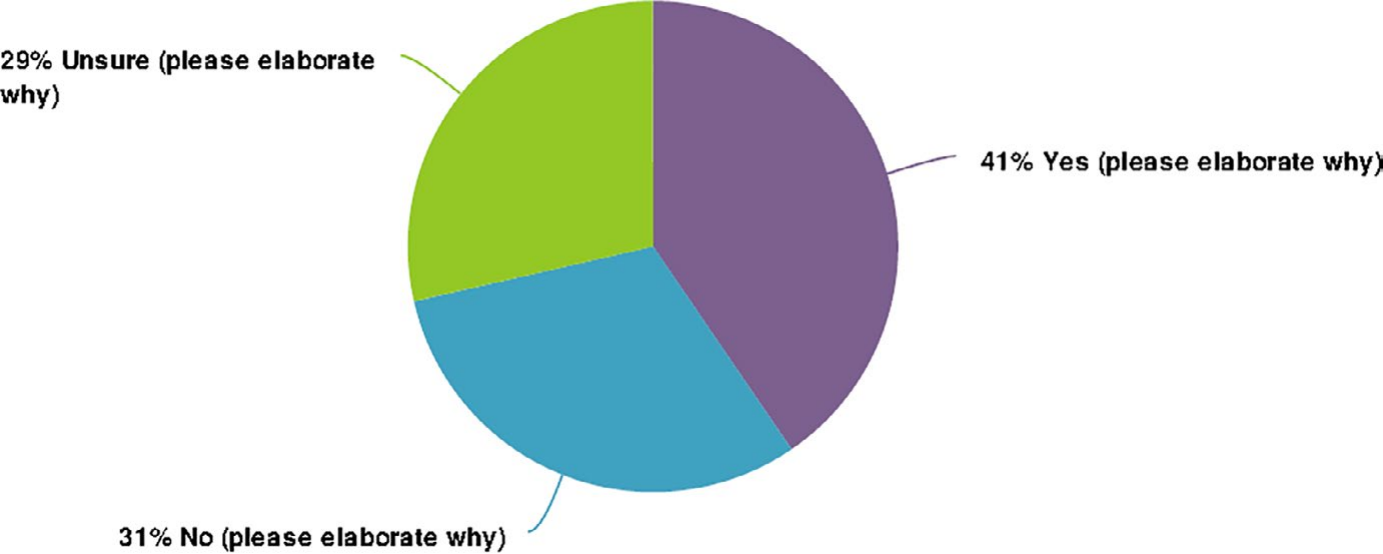
Confidence levels (survey question 29)

Responses to Q12 (Table 2) regarding confidence levels resulted in 38 (16%) respondents lacking confident in effectively supporting the preterm babies’ needs (Completely disagree 4.8%, Disagree 33.3%), with 6 (14.3%) being “Unsure.” This is further supported by both Q29 (Figure 3) in relation to how confident they were in their ability to care for the preterm baby and family and more substantively by Q18, with 18 (44%) and 9 (22%) responses to “Agree” and “Completely agree” respectively for the statement, “I need greater confidence to equip me to support parents of growing premature babies.” Participant 12 identified the challenges they experienced particularly with their confidence due to limited experience in the field of community practice with these families: “*Technologies and medical information is changing day to day, parents and their babies are all different so as best I can, I'm building confidence, however information is always changing*” (P12).

### 5.3 Existing knowledge

Some existing knowledge was confirmed in relation to short‐ and long‐term outcomes for preterm babies relating to long‐term respiratory issues, developmental and growth delay. Question 12 also revealed 8 (19%) and 71(6.7%) responses for “Agree” and “Completely agree” for adequacy of knowledge about the needs of preterm babies, 15 (35.7%) were “Unsure” and 1 (2.4%) and 11 (26.2%) answered “Completely disagree” and “Agree,” respectively. A very similar picture was seen for the question about knowledge on support for parents with 15 (36%) and 9 (22%) answering “Agree” and “Completely agree,” 15 (37.5%) were “Unsure” and 1 (2.4%) and 9 (22%) responded “Completely disagree” and “Agree,” respectively. Knowledge was obtained from varying sources including hospital policies, study days and, to concur with an earlier point, from nurse educators. The internet was also a common source of support with 24 (56%) participants stating this, due to the fact that “*most education takes place in capital cities with nothing offered locally*” (P3).

### 5.4 Previous training

Q13 asked about previous training in the specific needs of preterm babies and families—12 (28.5%) had a day or less, 10 (24%) less than a week and 10 (24%) greater than 2 weeks which was usually a placement within their initial nurse and/or midwifery training, for example on a neonatal unit. Three respondents (7%) had not received previous training. Adequacy of previous training was overall deemed to be very limited as seen in Q18 whereby there were 15 (37%) and 7 (17%) responses to “Disagree” and “Completely disagree,” respectively, for the statement, “I have received adequate training in the specific support needs of parents of growing premature babies.” Within the open‐ended responses, it was identified that for some participants, the content within their course was limited: “*Not as much as I would like, my course covered very little and the information from health department was sporadic at best*” (P26) and “*The core education was minimal so trying to build on such a weak foundation, with everything else there is to know as a CFHN [child and family health nurse] is not easy*” (P10).

### 5.5 Learning/Knowledge needs

**FIGURE 4:**
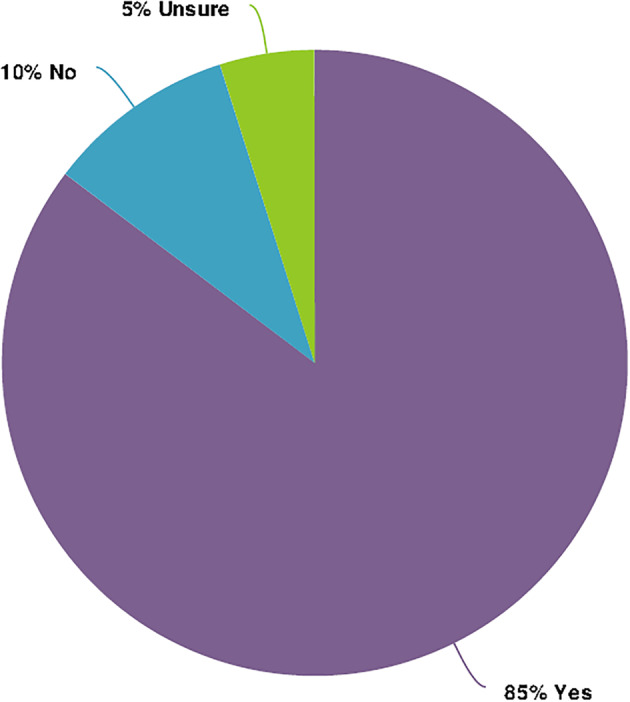
Whether more training is needed (survey question 15)

Related to the limited previous training received, responses to question 15 highlighted that the majority of participants felt more training was needed (Figure 4). Q18 provides further support to this finding, with 21 (50%) and 14 (34%) responses to “Agree” and “Completely agree,” respectively, for the statement, “I need more knowledge to equip me to support parents of growing premature babies.” Some participants identified that more knowledge was needed in regard to the growing premature baby in the open‐ended response questions: “*Long term outcomes, supporting families through a very traumatic time*” (P12) and “*I would enjoy learning about their milestones, what does corrected age mean and how does that impact in terms of expectations and care, what are the expected differences between premature and normal term babies. Just everything please*” (P33).

This knowledge need was identified for a variety of specific topics concurring with previous research (Green et al., 2020), as follows in order of prevalence:

1. Growth and Development
2. Recognizing when the premature baby is sick
3. Technology in the home (oxygen, feeding tubes, monitoring devices)
4. Availability and contacts for peer groups
5. Availability and contacts for counselling services
6. Dealing with common illnesses
7. Feeding
8. When and how to refer the baby when concerned
9. Settling

Thirty‐two (77%) responded that there was a need to integrate these topics into their professional training, 19 (45%) as a formal programme of study postqualifying and 24 (58%) stated this should also be combined with self‐directed study. One participant stated that she required: “*yearly, day long updates to keep my knowledge current & for me to best support families*” (P39).

### 5.6 Required resources

**FIGURE 5:**
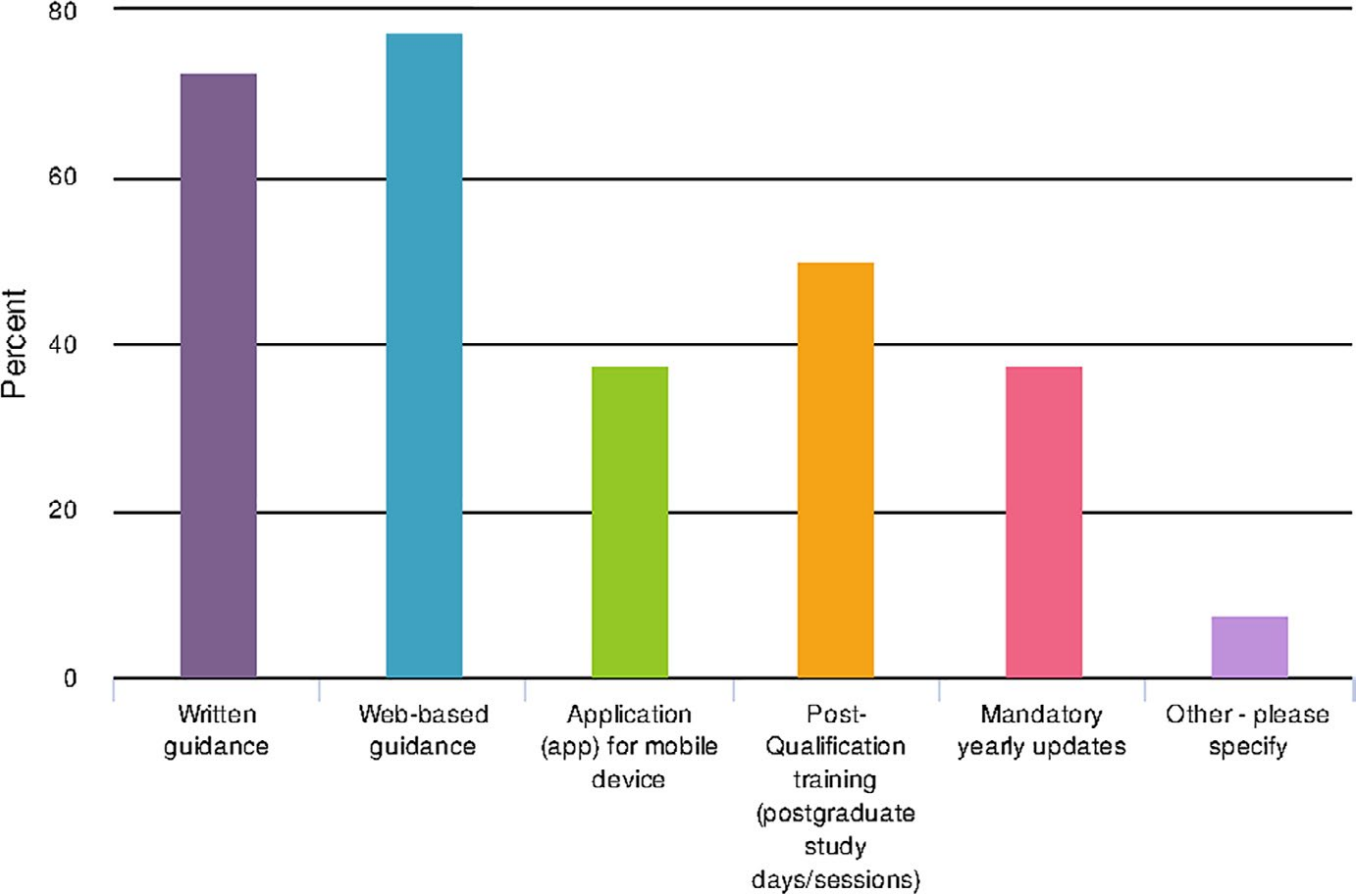
Resources required (survey question 20)

A large proportion (32 (77%)) of the participants responded “No” to Q26 when asked whether they had any special educational resources for the parents of premature babies. The same finding was present for question 20 regarding web‐based guidance as the most preferred resource for the participants to understand the specific needs of the premature baby (Figure 5) along with mobile phone applications combined with mandatory updates and postqualification study days. The need for “*reputable and evidence‐based online resources*” (P3) was highlighted and “*having access to colleagues and health professionals*” (P3) for additional support.

### 5.7 Barriers to provision of education

A key barrier to postqualification training and study days was the cost with 85% identifying this when asked about the factors that nurses face that stop them undertaking further education and professional development relating to the care of preterm babies. Other issues were lack of time (36 (80%)), lack of manager support (19 (46%)) and limited or no education in existence (25 (61%)). Within the open‐ended question responses received about additional issues related to face‐to‐face course attendance especially for nurses working in rural and isolated areas. For example: “*Distance, cost of travel, accommodation, unpaid leave, meals, course itself ‐ you end up looking at hundreds even, thousands of dollars*” (P33).

## 6 DISCUSSION

It is clear that caring for the preterm baby and family is associated with a specific set of knowledge and learning needs for community‐based nurses who care for them at home, due to the unique nature of their condition, physiology and ongoing issues that arise from being born prematurely. While the majority of nurses confirmed they were confident in their knowledge and skills related to the care of preterm infants, this was countered by nurses identifying that more training was needed. A reason provided for this was the changing knowledge about preterm infants and their care. This was noted as posing a challenge in maintaining competence levels.

Of note, was the high proportion of respondents stating that there was limited or no education available. This is concerning as with the development of new innovations in the care of preterm infants there is likely to be an increase in infant survival and need for long‐term community care. Gestation has a huge impact on survival and some babies require surgery, while others may have suffered from complications due to premature birth, for example an intraventricular haemorrhage. Despite the fact that both obstetric and neonatal healthcare teams may counsel against saving pre‐viable babies, because infants born at less than 23–24 weeks’ gestation are at highest risk of death or major morbidity (Anderson et al., 2016), the survival of babies born at extreme prematurity is becoming more common (Lantos, 2021); however, the immaturity of their organs will leave many of them with lifelong impairments necessitating ongoing care from the healthcare team (Ancel et al., 2015). An individualized approach to their ongoing recovery is required, and this is where the education of relevant health professionals such as community‐based nurses and other members of the primary healthcare team can make a real difference.

It appears that tailored education and training are required not only to address these nuanced features of prematurity but also in relation to the training gap that is evident. An increase in the content in postgraduate programmes, that prepare nurses to work in the community, is essential as this is currently lacking, confirmed by recent evidence that has reported a lack of tailored education for community‐based nurses (Petty et al., 2018; Malone et al., 2015 cited by Green et al., 2020; Petty, Whiting, et al., 2019). It was the analysis from these studies that led to the awareness of the need to undertake more research in knowledge and skill base for community‐based nurses and what education is required. Leading from this, is the question of what is the best approach to provide such education?

Literature has highlighted various strategies with suggestions for what is useful within the above context. An early study by Pridham et al. (2006) advocated specific role enhancement (in the form of a primary health nurse) and a need for a long intervention period, in the context of interdisciplinary and interagency collaboration. Although undertaken 15 years ago, it was only much later that Burns et al. (2017) supported these suggestions, stating the necessity for facilitating access to existing community resources by linking individuals to services, including signposting, referral or facilitation to engage with initiatives that go beyond traditional health approaches. Workshops have also been suggested as an effective way for community health professionals to, for example, enhance knowledge of lower respiratory tract infections (Kamil‐Thomas, 2018).

Resource development, that is tailored to the specific needs of preterm babies and families, is also important, concurring with our earlier UK‐based study (Petty, Whiting, et al., 2019). Websites with helpful materials to organize problem lists and follow up visits that are readily accessible have been suggested by VenOsdel et al. (2015). This may address the barriers identified by the participants in this study. Indeed, an impediment for some nurses are the barriers to accessing ongoing professional development. These challenges can be exacerbated for nurses working in countryside and isolated areas. However, in a recent study, nurses working in Chinese rural facilities were found to have very positive attitudes towards e‐Learning (Xing et al., 2020). Therefore, an online approach to resource development can enable a wider audience and reach, addressing problems of distance and access to education programmes.

As an outcome of the 2020 COVID‐19 pandemic, there has been a dramatic increase in the use of digital technologies for clinical practice and education. Nurses are developing increased acceptance and competence in using these technologies to access ongoing education (Bennett et al., 2020). This supports the concept that the more nurses use computers, the more they increase their acceptance of online resources (Xing et al., 2020) including social media.

The use of social media in this study raises an important discussion point relevant to education. As outlined in the methodology section, Facebook groups associated with professional organizations were used as a platform to recruit the participants as they provided easy and cost‐effective access to large numbers of people across wide geographic boundaries (Korda & Itani, 2014), in this case within Australia. However, it is noteworthy that many Facebook groups focus on preterm babies, largely comprising parents of preterm babies, particularly mothers. They use these forums for information sharing (Thoren et al., 2013) about the ongoing care of their baby, and to receive emotional support from other mothers in the same situation. Health professionals should be aware that social media sites serve as sources of healthcare information; Facebook is often the preferred site for those seeking information (Park et al., 2011). People are increasingly using the internet; studies have shown that it is widely used to find health‐ and disease‐related information more frequently than communication with a doctor (Greene et al., 2010). One caution is that Facebook groups depend on crowd‐sourced information by enlisting services of many people and rely on the connected Facebook users to access and provide the information (Gage‐Bouchard et al., 2018); this, however, might not be scientifically validated or evidence‐based, and health professionals have concerns about the spread of misinformation on social media. Antheunis et al. (2013) have emphasized that health professionals need to grasp the potential of social media and positively embrace it, to provide trustworthy information. To gain an authentic understanding of the ongoing needs of premature babies and their families, the authors propose that it may be useful for community‐based nurses to join the premature baby support groups on platforms such as Facebook.

### 6.1 Implications for future practice

**FIGURE 6:**
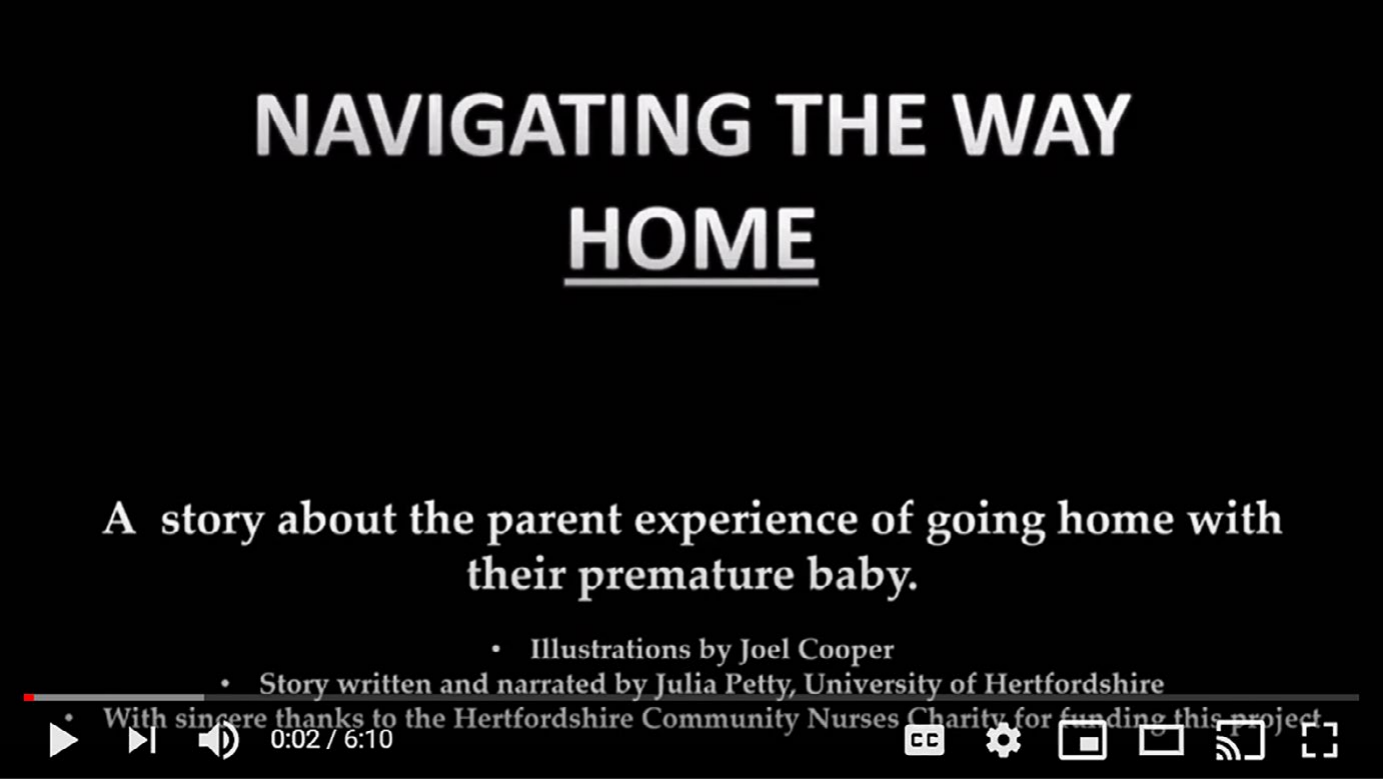
Digital story

The study findings support the need for development of tailored resources delivered on an online platform to ensure reach and accessibility. Examples are illustrated by a web‐based platform developed by the lead author of this paper, hosting a collection of tailored information on the needs of the premature baby (https://neonatalstories.com/parent‐stories/). In addition, a digital story has been developed: *Navigating the Way Home* https://www.youtube.com/watch?v=sWnc8P‐yaPA&feature=youtu.be (Figure 6) that focuses on the issues relating to the transition home and includes signposting to relevant resources.

Ensuring community‐based nurses are aware of the benefits of such learning resources is essential to enhance knowledge and optimize the potential that technology offers. Some nurses have been found to be reluctant to engage with online learning due perhaps to its perceived effectiveness being relatively low (Karaman, 2011) and/or integrating inadequate communication, support and interaction (Bramer, 2020). Furthermore, a study by Mackay et al. (2017) of mobile devices used to support clinical teaching, identified that adequate resourcing and technology advice was needed to provide community‐based nurses with the necessary support. The resources developed and signposted here are a start. There remains a need for further evidenced‐based educational resources for community nurses involved in caring for preterm babies and their families at home, on a number of different topics, such as developmental milestones, feeding, weight gain and growth, recognizing the deteriorating baby and emotional support of parents. This paves the way for future educational resource development.

### 6.2 Limitations

Finally, the study limitations are recognized to ensure transparent and balanced reporting. Firstly, the sample size was small and did not reflect a range of roles, ethnicities or genders (although it is important to note that nursing remains a female dominated profession), which may have led to bias; the inclusion criteria were therefore, perhaps too restrictive. Further roles would be interesting to explore relating to other primary care health professionals, indicating again potential future work with a larger sample and broader inclusion. Secondly, some participants did not complete the full survey which may have affected accuracy of reporting in relation to incomplete data (McInroy, 2016). It is not known why this was the case, but a possible reason could be that the survey design was not sufficiently clear with further instruction required to participants to complete both parts. The survey could also have been too lengthy, taking too much time to complete. Literature states these issues need considering with surveys (Coste et al., 2013), along with the need to externally pilot them before use and prevent participants being able to repeat taking part, limitations previously acknowledged in the Methods section. The study's limitations may have potentially affected the reliability and validity of the findings (Bennett et al., 2010). However, it can be argued that the online survey approach does have transferability to other countries where remote residences and long distances are an issue. Nonetheless, it was essential that the researchers adhered to established guidelines for survey reporting (Kelley et al. (2003).

## 7 CONCLUSION

This study explored perceived knowledge, confidence levels and learning needs of community‐based nurses in relation to caring for this specific group of babies and their families. The findings identified and informed what is required to equip them with the knowledge and skills to support parents following discharge home from the neonatal unit. This is important as more very preterm babies are surviving and going home, meaning that nurses working in the community will potentially be caring for these families on a more regular basis. Surviving premature babies have ongoing health issues that need to be met by professionals who are educated and understand the complexity of developmental differences between them and fullterm babies. While it is true that parents become the experts in their babies, they rely on appropriately educated health professionals to help them navigate the healthcare system.

## CONFLICT OF INTEREST

There is no conflict of interest to declare for any of the named authors above.

## AUTHOR CONTRIBUTIONS

All authors contributed to the study design, undertaking the data collection, analysis and the report write‐up.

## Data Availability

Data available on request from the authors / The data that support the findings of this study are available from the corresponding author upon reasonable request.
